# A multi-view CNN model to predict resolving of new lung nodules on follow-up low-dose chest CT

**DOI:** 10.1186/s13244-025-02000-x

**Published:** 2025-06-27

**Authors:** Jingxuan Wang, Xiaowen Zhang, Wei Tang, Marcel van Tuinen, Rozemarijn Vliegenthart, Peter van Ooijen

**Affiliations:** 1 Department of Radiology, University of Groningen, University Medical Center of Groningen, Groningen, The Netherlands; 2 Data Science in Health (DASH), University of Groningen, University Medical Center of Groningen, Groningen, The Netherlands; 3 Department of Neurology, University of Groningen, University Medical Center of Groningen, Groningen, The Netherlands; 4 Department of Radiation Oncology, University of Groningen, University Medical Center of Groningen, Groningen, The Netherlands

**Keywords:** Pulmonary nodule, Computed tomography, Deep learning, Convolutional neural networks

## Abstract

**Objective:**

New, intermediate-sized nodules in lung cancer screening undergo follow-up CT, but some of these will resolve. We evaluated the performance of a multi-view convolutional neural network (CNN) in distinguishing resolving and non-resolving new, intermediate-sized lung nodules.

**Materials and methods:**

This retrospective study utilized data on 344 intermediate-sized nodules (50–500 mm^3^) in 250 participants from the NELSON (Dutch-Belgian Randomized Lung Cancer Screening) trial. We implemented four-fold cross-validation for model training and testing. A multi-view CNN model was developed by combining three two-dimensional (2D) CNN models and one three-dimensional (3D) CNN model. We used 2D, 2.5D, and 3D models for comparison. The models’ performance was evaluated using sensitivity, specificity, and area under the ROC curve (AUC). Specificity, indicating what percentage of non-resolving nodules requiring follow-up can be correctly predicted, was maximized.

**Results:**

Among all nodules, 18.3% (63) were resolving. The multi-view CNN model achieved an AUC of 0.81, with a mean sensitivity of 0.63 (SD, 0.15) and a mean specificity of 0.93 (SD, 0.02). The model significantly improved performance compared to 2D, 2.5D, or 3D models (*p* < 0.05). Under the premise of specificity greater than 90% (meaning < 10% of non-resolving nodules are incorrectly identified as resolving), follow-up CT in 14% of individuals could be prevented.

**Conclusion:**

The multi-view CNN model achieved high specificity in discriminating new intermediate nodules that would need follow-up CT by identifying non-resolving nodules. After further validation and optimization, this model may assist with decision-making when new intermediate nodules are found in lung cancer screening.

**Critical relevance statement:**

The multi-view CNN-based model has the potential to reduce unnecessary follow-up scans when new nodules are detected, aiding radiologists in making earlier, more informed decisions.

**Key Points:**

- Predicting the resolution of new intermediate lung nodules in lung cancer screening CT is a challenge.
- Our multi-view CNN model showed an AUC of 0.81, a specificity of 0.93, and a sensitivity of 0.63 at the nodule level.
- The multi-view model demonstrated a significant improvement in AUC compared to the three 2D models, one 2.5D model, and one 3D model.

**Graphical Abstract:**

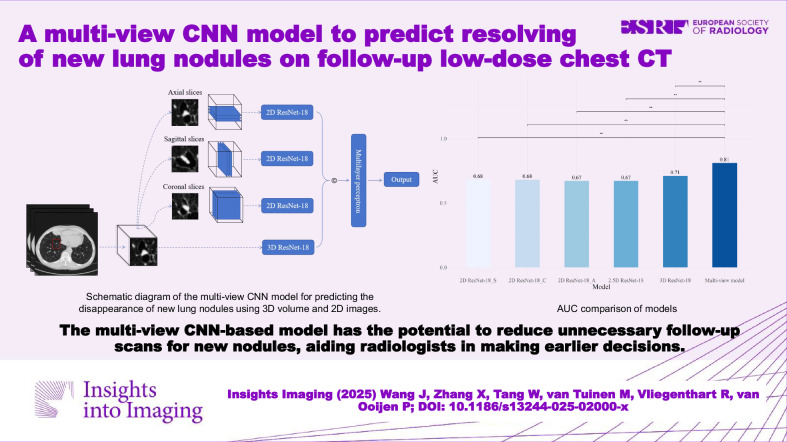

## Introduction

Screening trials, including the Dutch-Belgian Randomized Lung Cancer Screening Trial (NELSON), demonstrated a significantly lower lung cancer mortality in high-risk individuals who underwent low-dose computed tomography (LDCT) lung cancer screening than in those without screening [1]. During each incidence screening round for lung cancer, newly developed nodules were found in 5–7% of screening participants in the NELSON study [2, 3]. Compared to nodules detected at baseline screening, new nodules develop over a short time and are more likely to resolve. In two incidence screening rounds from NELSON, about 55% of new nodules disappeared at subsequent scans [4], while 4% of new nodules were lung cancer [3]. In lung cancer screening, false positives may cause unnecessary imaging examinations or work-ups [5, 6]. The National Lung Screening Trial (NLST) study showed that about 23% of participants had false-positive results in the three LDCT screening rounds. Other potential risks of LDCT screening include unnecessary invasive interventions, overdiagnosis, and radiation-induced cancers. Currently, management regarding follow-up CT is primarily based on nodule size and appearance. Given the advances in nodule evaluation and cancer diagnosis using deep learning (DL), DL techniques could potentially be a complementary method for reducing unnecessary follow-up LDCT scans [7–9].

Convolutional neural networks (CNN) show promising performance in lung nodule research [10]. Setio et al [11] proposed a multi-view CNN that effectively differentiated between true nodules and false positives by using nine two-dimensional (2D) CNNs, each corresponding to one of the 2D views extracted from nine oriented planes, with the final output generated through a late-fusion strategy. Such multi-view strategies and their variants were also applied to classify benign and malignant nodules [12, 13]. To include spatial information, three-dimensional (3D)-based CNN models have been used for various tasks, such as nodule classification [14] and malignancy risk prediction [15]. Venkadesh [16] developed an ensemble of nine 2D ResNet-50 and one 3D Inception-v1 to predict nodule malignancy risk, reaching an area under the receiver operator characteristic curve (AUC) of 0.93. Recently developed DL models have incorporated more transparent visualization methods [17], such as Grad-CAM and Grad-CAM++. These methods highlight the image regions that contributed most to the model decision, thereby enhancing model explainability.

To our knowledge, this is the first study to predict the disappearance of new nodules identified during follow-up screening rounds using an explainable CNN model based on LDCT imaging data. The final aim of this study was to evaluate the CNN model’s efficacy in predicting the resolution of new intermediate-sized solid nodules on follow-up screening LDCT scans.

## Materials and methods

### Data collection and selection

**Fig. 1:**
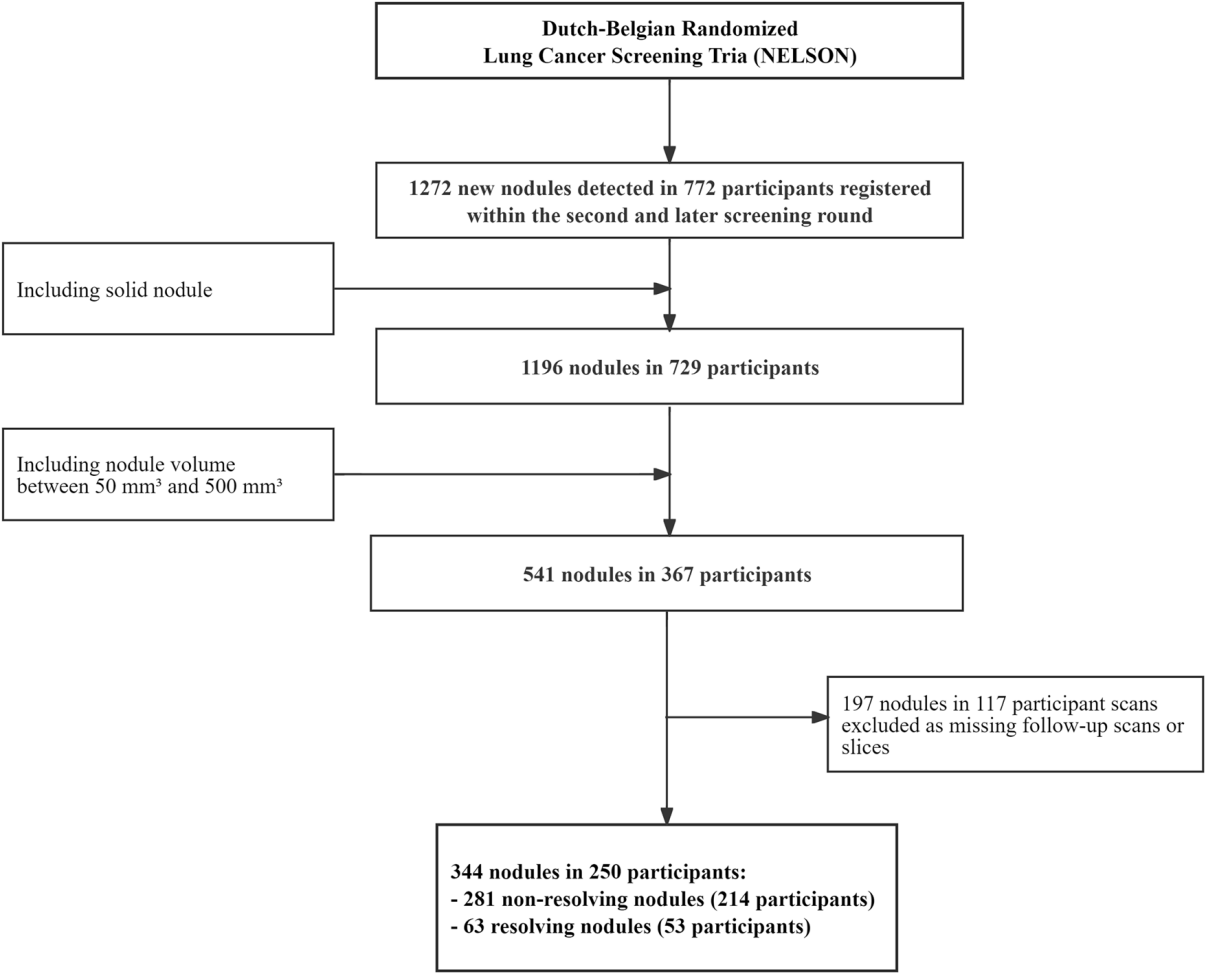
Flowchart of the data selection of new lung nodules in the NELSON

We obtained access to a dedicated selection from NELSON for this study after data use approval from the NELSON board. Details on the NELSON study design have been published [18]. In short, screening participants received baseline LDCT scans from 2004 to 2006 in four medical centers. Regular follow-up screenings were performed at years 1, 2, and 5.5 after baseline and short-term follow-up scans in case of intermediate-sized nodules. Inclusion criteria for the current analysis included the following: (1) nodules registered as new by radiologists on incidence screening rounds, with a next follow-up scan available; (2) solid nodules; (3) nodules with intermediate size (50–500 mm^3^). Participants with missing scans or incomplete scan slices were excluded from the analysis. The details for inclusion and exclusion are outlined in Fig. 1. Nodules classified as resolving were those that disappeared at follow-up scans, and those that were in the process of resolving and no longer had solid components.

### Training and validation sets

To meet the input requirements of the CNN model, nodule annotation and data splitting were conducted sequentially. The nodule was labeled in 3D Slicer by marking its approximate centroid on the CT scan, with detailed annotation example images provided in our previously published paper [19]. A thoracic surgeon (X.Z., with three years of chest CT reading experience) annotated the coordinates of nodules based on previously recorded nodule metadata. Potential annotation discrepancies were reviewed by an experienced radiologist (R.V., with 19 years of chest CT reading experience). The dataset, comprising a collection of new nodules, was divided into four folds using a stratified four-fold validation approach. The original category proportions were maintained in each fold. In total, four groups of training and test sets were generated (see Fig. A1 in Appendix A). The training set of each group included the nodules from three folds, and the remaining one was an internal validation set.

### Image preprocessing

The lung window was adjusted to optimal evaluation settings [WW: 1600 HU, WL: −700 HU] for nodules. We used the B-spline interpolation technique to interpolate LDCT volumes to a voxel size of 1 × 1 × 1 mm, ensuring uniformity in all scans. The lung nodules were extracted from the LDCT images and saved into a cubic centroid (32 × 32 × 32 mm^3^). The 3D image box of the nodule was placed such to preserve its complete spatial information, which is not available in 2D images. To acquire the most representative nodule information, nine 2D images were extracted from the 3D volumes, three on each of the image planes (coronal, sagittal, and transverse planes), with the center point located in the middle of the nodule.

### Multi-view convolutional neural network

**Fig. 2:**
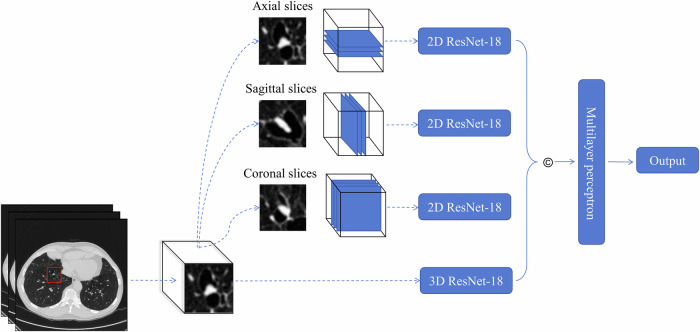
Schematic diagram of the multi-view CNN model for predicting the disappearance of new lung nodules using 3D volume and 2D images. Four views of the nodules are processed by four ResNet-18 models. The extracted features from each model are concatenated (©) and then passed through a multi-layer perceptron to make the final prediction

We proposed a multi-view CNN model for capturing the features of nodules in 3D images and 2D images, as illustrated in Fig. 2. The model architecture was composed of four primary components: three 2D ResNet-18 modules and one 3D ResNet-18 module.

Each 2D ResNet-18 network was designed to process three consecutive middle slices of 3D nodules along specific anatomical axes—axial, coronal, and sagittal. These slices were concatenated along the channel dimension to create a three-channel input. The 3D ResNet-18 network processed the volumetric data of the 32 × 32 × 32 mm^3^ cubic centroid. The outputs of the four networks (three 2D and one 3D) were concatenated to form a 72-dimensional feature vector, which was then fed into a multi-layer perceptron to produce the final class probabilities. This multi-view approach combines detailed sectional information from 2D views with spatial relationships from 3D volumes to enhance prediction accuracy. More details are described in Appendix A2.

To evaluate the proposed multi-view model, we compared its performance against five sub-models trained and validated independently. These comparison experiments considered variations in different views and dimensions of the nodules and models: (1) 2D ResNet-18_A, processing axial slices; (2) 2D ResNet-18_C, processing coronal slices; (3) 2D ResNet-18_S: a 2D processing sagittal slices; (4) 2.5D ResNet-18, integrating the outputs of the three 2D ResNet-18 models; and (5) 3D ResNet-18, analyzing volumetric data in three dimensions. More information about the experimental settings is shown in Appendix A3.

### Model explainability

Given the successful use of interpretability methods in CNN models for image classification, we used Grad-CAM++ to understand model predictions. This method creates visual explanations for a specific class label by using a weighted combination of positive partial derivatives from the feature maps in the last convolutional layer [20]. From a visual interpretability perspective, Grad-CAM++ produced heatmaps to localize and highlight the most influential regions of resolving or non-resolving nodules that impact the model’s predictions.

### Evaluation metric and statistical analysis

Model predictions were evaluated using sensitivity, specificity, and AUC. In this study, specificity evaluated what percentage of non-resolving nodules that require follow-up were identified correctly. Sensitivity evaluated how many resolving nodules were identified correctly. We aimed to maximize specificity, as conducting a follow-up scan for a resolving nodule is not a clinical problem. However, in the context of screening, it is important not to miss follow-up CT having intermediate nodules that do not resolve and may potentially indicate lung cancer. The sensitivity and specificity from four-fold cross-validation were reported as mean values with corresponding standard deviations (SD). We also calculated the participant-based results (the set of rules is shown in Appendix A4), showing what percentage of scans could be prevented. *p*-values were calculated for AUC comparisons to assess the statistical significance of differences between models. As of the last follow-up, we observed that ten nodules had not completely disappeared but were in the process of resolving and lacked solid components. To explore the potential impact of those (“nearly-resolved”) nodules on our analysis, we performed a sub-analysis where these ten nodules were excluded. The 95% confidence intervals (CIs) and *p*-values for the AUC values were computed using the pROC package (R version 4.3.2), with the DeLong method employed to compare AUCs. Statistical significance was defined as *p* < 0.05.

## Results

### Dataset characteristics

The dataset contained 344 new intermediate nodules in 250 participants (54 women, 21.6%) with follow-up scans. The median age was 60 years [interquartile range (IQR), 56–64]. The median smoking history was 38.7 pack-years [IQR, 29.7–53.2]. Most of the participants were current smokers (58.1%, 140/241, information in 9 participants missing). Nodule characteristics are provided in Table 1.

**Table 1:** Initial characteristics of new intermediate-sized nodules

	Non-resolving nodules (*n* = 281)	Resolving nodules (*n* = 63)	*p*-value
Volume			0.30
Median (IQR)	127.6 (82.1, 236.9)	129.5 (77.6, 195.6)	
Maximum diameter			0.36
Median (IQR)	8.4 (6.9, 11)^a^	8.4 (6.9, 9.7)^b^	
Minimum diameter			0.39
Median (IQR)	5.3 (4.4, 6.9)^a^	5.6 (4.5, 6.7)^b^	
Edge			0.39
Smooth	244 (88.4%)^c^	50 (83.3%)	
Non-smooth	32 (11.6%)^c^	10 (16.7%)	

*p*-values are calculated using the Mann–Whitney U tests for numerical data and the Chi-square test for categorical data

^a^ The medians and IQR calculations were based on 278 nodules because 3 nodules lacked data on maximum and minimum diameter

^b^ The medians and IQR calculations were based on 61 nodules because 2 nodules lacked data on maximum and minimum diameter

^c^ The percentages for non-resolving nodules were based on 276 nodules because 5 nodules lacked data on edge. The percentages for resolving nodules were based on 60 nodules because 3 nodules lacked data on edge

### Algorithm performance

**Fig. 3:**
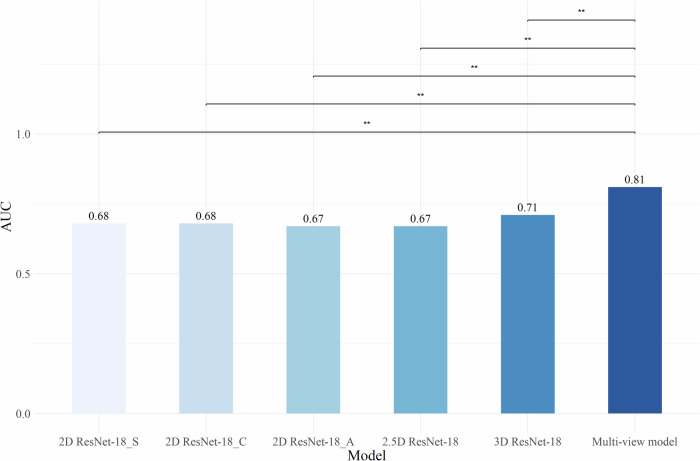
AUC comparison of models (multi-view model is built on three 2D models and one 3D model. A: axial view; C: coronal view; S: sagittal view; **: significant at *p* < 0.01)

Figure 3 presents the AUC comparison between the proposed multi-view model and the other five models (0.81 [0.75, 0.88] vs 0.68 [0.61, 0.75], 0.68 [0.60, 0.76], 0.67 [0.59, 0.75], 0.67 [0.59, 0.75], 0.71 [0.63, 0.79]). The proposed model showed significantly higher AUC than other models, with *p*-values consistently below 0.01. Table 2 displays the sensitivity and specificity from the 4-fold cross-validation of each model.

**Table 2:** Sensitivity and specificity of 4-fold cross-validation on nodule basis

	Sensitivity	Specificity
2D ResNet-18_A	0.46 ± 0.07	0.89 ± 0.05
2D ResNet-18_C	0.49 ± 0.10	0.83 ± 0.05
2D ResNet-18_S	0.49 ± 0.07	0.81 ± 0.07
2.5D ResNet-18	0.48 ± 0.13	0.86 ± 0.11
3D ResNet-18	0.57 ± 0.12	0.84 ± 0.04
Multi-view model	**0.63** ± 0.15	**0.93** ± 0.02

Multi-view model is built on three 2D models and one 3D model. The results are the mean ± standard deviation of 4-fold cross-validation. A: axial view; C: coronal view; S: sagittal view; the best result is in bold, and the second-best result is underlined

**Fig. 4:**
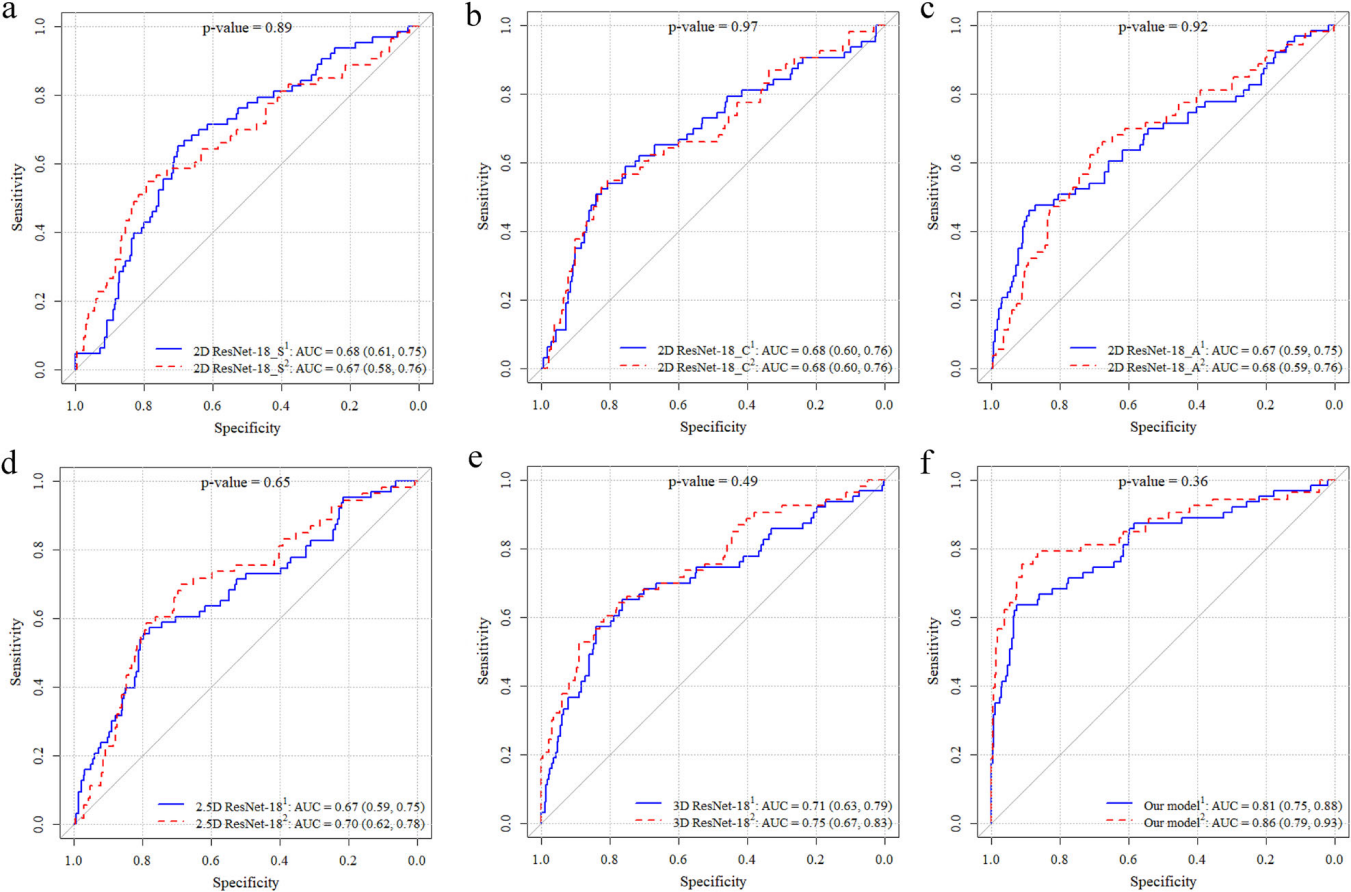
ROC showing model performance on data where ten nearly-resolved nodules were either included or excluded (**a**–**f** represent six model comparisons in two datasets; ^1^: the model was tested in the dataset including nearly-resolved nodules, blue; ^2^: the model was tested in the dataset excluding nearly-resolved nodules, red)

Figure B1 and Table B2 in Appendix B (Electronic Supplementary Material) present the AUC and sensitivity and specificity of the models tested on the dataset, excluding ten nearly-resolved nodules. Figure 4 not only shows the AUC for each model but also shows the potential impact of nearly-resolved nodules on model learning. All *p*-values were above the 0.05 significance threshold (*p* = 0.89, 0.97, 0.92, 0.65, 0.49, 0.36), suggesting that the addition of nearly-resolved nodules for each model did not significantly alter performance.

### Explainable result

**Fig. 5:**
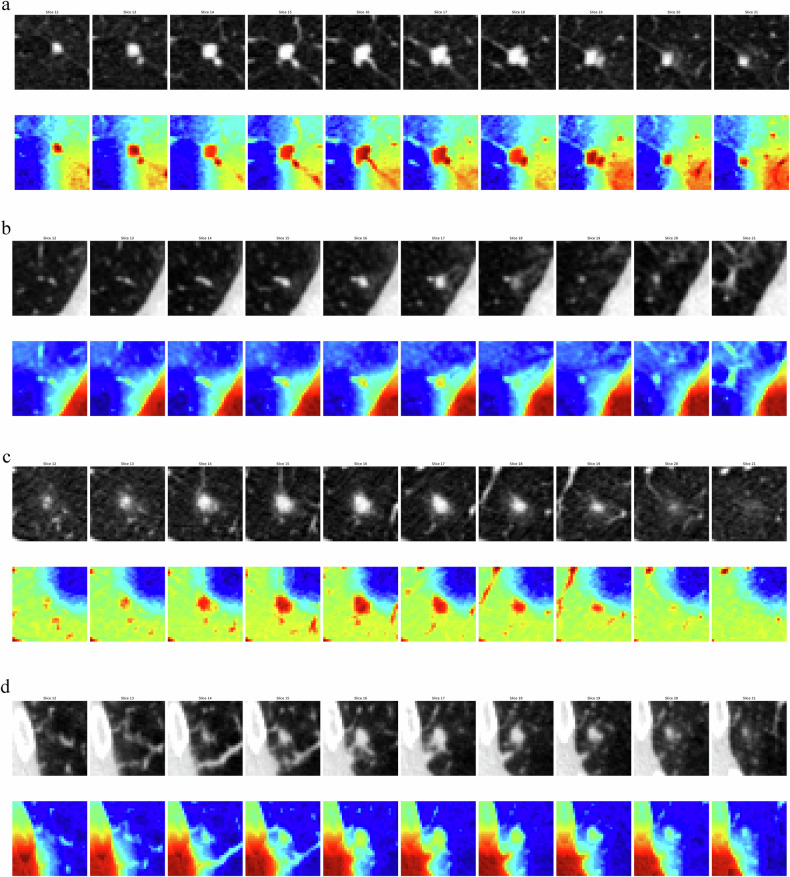
Four examples of resolving nodules and non-resolving nodules (first row of each image is the original image; second row is the overlaid image; **a** true prediction of non-resolving nodule; **b** false prediction of non-resolving nodule; **c** true prediction of resolving nodule; **d** false prediction of resolving nodule)

Grad-CAM++ was employed to visualize the regions of interest that contributed to the model’s predictions regarding the disappearance of pulmonary nodules. The generated heatmaps, overlaid on the CT images, highlight the areas of the nodules that the deep learning model considered significant for prediction. The red areas represent the regions where the model assigned the highest importance, and the yellow areas indicate moderately important regions (as shown in Fig. 5).

### Participant-based calculation

In our study, 53 participants had resolving nodules. Among them, 17 participants had resolving and non-resolving nodules, while the remaining 36 participants had only resolving nodules. The participant-based outcomes are shown in Table B3. Our multi-view model predicted 35 positive cases (the model predicted that intermediate-sized nodules would resolve), correctly identifying 63% (22/35) participants with only resolving nodules. Additionally, the model demonstrated a high participant-based specificity of 0.94 and a sensitivity of 0.61. In the entire dataset analyzed, 14% (35/250) of participants could avoid follow-up scans, at the expense of 37% (13/35) inadvertently having a non-resolving nodule.

## Discussion

In this study, we developed and evaluated a multi-view convolutional neural network (CNN) to predict the resolution of new intermediate-sized nodules in lung cancer screening. The proposed multi-view model achieved an AUC of 0.81 with a mean specificity of 0.93 (SD, 0.02).

The multi-view CNN model achieved the highest AUC compared to individual 2D, 2.5D, and 3D models, suggesting that the combination of 2D and 3D models leads to improvement in discriminatory performance compared to the individual models. The rationale of designing a multi-view model assumes that leveraging several 2D ResNet-18 models can learn distinct characteristics of nodules from different views. The axial, sagittal, and coronal views offer complementary features, and the three middle slices with the largest diameters provide the most representative views. Furthermore, we enhanced the richness of the nodule’s representation by combining these three greyscale images into a single RGB. The 3D ResNet-18 model is capable of learning spatial information across the entire volume of the nodules, thus compensating for the information that the 2D model might miss. However, particularly for small nodules, the 3D nodule block contains a larger portion of background tissue than the larger nodules. This excess non-nodule space can introduce noise, which may diminish the model’s learning capacity. Our proposed model leverages the advantages of both 2D and 3D approaches, combining the complementary strengths of each to improve nodule prediction. From a clinical perspective, while radiologists can assess the characteristics of pulmonary nodules, they often cannot reliably predict the likelihood of nodule resolution, making models like ours valuable in providing objective insights to support their clinical decision-making. A similar study also demonstrated the effectiveness of the 2D and 3D ensemble model; a prediction DL model proposed by Venkadesh showed a superior performance (AUC, 0.93 vs 0.90 in the clinical model) in assessing the malignancy of lung nodules [16].

To implement the model in a clinical setting, it is crucial to ensure high specificity, which in this study is defined as the percentage of correctly identified non-resolving nodules. Non-resolving nodules may exhibit growth during follow-up examinations, and false predictions of them can lead to missed assessments of these nodules. The current model correctly predicted 63% of predicted positive cases, which should be further developed and optimized for the model to be used in clinical practice. The multi-view model achieved more than 90% specificity, outperforming the other five models evaluated. Once high specificity is achieved, improving model sensitivity can be considered. A low sensitivity level does not pose a significant risk for clinical use of the model, as resolving nodules predicted as non-resolving will still undergo subsequent follow-up examinations. Participants with at least one non-resolving nodule should still undergo follow-up rounds to monitor changes in these nodules. At the participant level, our model demonstrated the potential to reduce follow-up scans by 14%, while maintaining a sensitivity of 0.61 and a high specificity of 0.94 in correctly identifying participants with non-resolving nodules. In our study, resolving new intermediate-sized nodules accounted for only 18% of the cases, representing a minority class. Therefore, the percentage of screening participants in whom unnecessary follow-up scans could be prevented was relatively low (14%). Furthermore, due to the large percentage of screening individuals with at least one non-resolving nodule, the proportion of false positives was relatively high (37%). This observation may reflect selection bias in our dataset, which likely led to an overrepresentation of non-resolving nodules compared to an unselected screening population. In a prior NELSON publication based on new nodule data from the second and third screening rounds, intermediate-sized nodules showed disappearance in 56% [4]. Thus, in a real-life setting, the percentage of screening participants in whom follow-up CT is unnecessary is likely higher, and the balance with false positives is more favorable.

Our proposed model showed no difference in performance in the dataset with and without nearly-resolved nodules. Given that our study only included 10 near-resolved nodules, the lack of a statistical difference does not necessarily imply that the model is incapable of distinguishing between resolving and nearly-resolved nodules. We can only conclude that no significant difference in model performance was observed between the two datasets. However, it remains uncertain whether this result would persist if more nearly-resolved nodules were added.

A limitation of this study is the lack of an external validation dataset. The models’ performance has been validated on the internal dataset. Given the nature of the nodules for our study, finding a suitable external dataset poses two challenges: it must include multiple rounds of follow-up CT scans and newly developed nodules in those follow-ups. To address this, we first considered that NELSON is a multi-center study, which may support the model’s generalizability to some extent. Second, we utilized cross-validation to maximize the use of our available dataset, providing a robust assessment of model performance when external validation is not feasible. We believe this approach still offers a reliable estimate of the model’s generalization ability. In the future perspectives, one is to improve the model performance on sensitivity; another is to collect the timing of nodule resolution as additional information for prediction.

In conclusion, our findings indicate that a multi-view CNN-based model could be a valuable tool in lung cancer screening by providing nodule disappearance predictions to assist radiologists in decision-making. This approach has the potential to reduce unnecessary follow-up CT scans by accurately predicting which nodules are likely to disappear.

## Supplementary information

ELECTRONIC SUPPLEMENTARY MATERIAL

## Data Availability

Access to data can be requested through the NELSON board.

## Abbreviations

2D: Two-dimensional
3D: Three-dimensional
AUC: Area under the receiver operating characteristic curve
CNN: Convolutional neural network
DL: Deep learning
IQR: Interquartile range
LDCT: Low-dose computed tomography
NELSON: Dutch-Belgian Randomized Lung Cancer Screening Trial
SD: Standard deviation

## Code availability

The code can be provided on request.
