# Supplementary material for: A multi-view CNN model to predict resolving of new lung nodules on follow-up low-dose chest CT
Source: Insights Imaging. 2025 Jun 27;16:138. doi: 10.1186/s13244-025-02000-x (PMC12205119; doi:10.1186/s13244-025-02000-x)
Supplement: Supplementary file 1 — ELECTRONIC SUPPLEMENTARY MATERIAL [file 13244_2025_2000_MOESM1_ESM.pdf]

# A MULTI-VIEW CNN MODEL TO PREDICT RESOLVING OF NEW LUNG NODULES ON FOLLOW-UP LOW-DOSE CHEST CT

# ELECTRONIC SUPPLEMENTARY MATERIAL

## Appendix A:

### 1 Four-fold cross-validation strategy

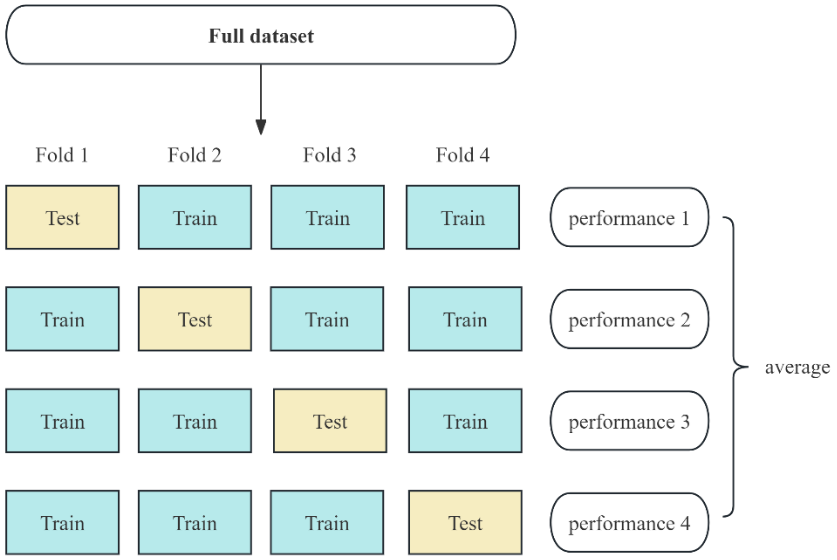


Figure A1 Training and testing sets of four-fold cross-validation. Each train/ test cell contained 86 nodules in total: 16 resolving nodules and 70 non-resolving nodules for Folds 1-3; 15 resolving nodules and 71 non-resolving nodules for Fold 4.

## 2 Model architecture

2.1 2D ResNet-18 network

2D ResNet-18 is a widely used CNN architecture originally designed for image classification tasks. It pioneered the use of "residual learning block" in deep networks, significantly enhancing accuracy while simultaneously reducing training complexity. The 2D ResNet-18 architecture consists of 18 layers. First, an initial 7x7 convolutional layer is applied, followed by a max-pooling layer. Next, four residual blocks are introduced, each containing two 3x3 convolutional layers with bypass residual connections. Subsequently, a global average pooling layer and softmax activation are applied. Finally, a fully connected (fc) layer outputs class probabilities.

Each 2D ResNet-18 network was adapted to deal with slices of the 3D nodules along a specific axis:

i. ResNet-18 network for axial slices processed three middle consecutive axial slices concatenated along the channel dimension to form a three-channel input (2D Resnet-18_A).
ii. ResNet-18 network for coronal slices similarly processed three middle consecutive coronal slices (2D Resnet-18_C).
iii. ResNet-18 network for sagittal slices similarly processed three middle consecutive sagittal slices (2D Resnet-18_S).

The pre-trained weights on the ImageNet dataset provided the initial parameters for model training. The final fully connected (fc) layer of each network was modified to output an 18-dimensional feature vector, optimizing its contribution to the fusion stage. For comparison purposes, the 2.5D model was constructed using 2D ResNet-18_A, 2D ResNet-18_C, and 2D ResNet-18_S Models. The final layer of the 2.5D network had 54-dimensional output feature vectors.

2.2 3D ResNet-18 network

3D ResNet-18 extends the ResNet-18 architecture into three dimensions, making it suitable for direct processing of volumetric data such as CT scans. The 3D ResNet-18 architecture comprises: (1) An initial 3D convolutional layer with 7x7x7 kernels; (2) Four 3D residual blocks, each containing two 3D convolutional layers with 3x3x3 kernels; (3) A 3D global average pooling layer; and (4) A final fc layer that outputs class probabilities.

In our study, the 3D ResNet-18 model was customized to process the entire nodule saved in 3D volume, with pre-trained weights serving as the starting point for model training. The final fully connected layer was modified to output an 18-dimensional feature vector, consistent with the 2D networks.

## 3 Experimental setting

Four-fold stratification was performed using the scikit-learn package (v1.3.2). During the training phase, data augmentation included random rotation (probability = 0.6, the range of rotation angle around the x-axis: [-0.4, 0.4] radians) and random flipping (probability = 0.6). Backbone models were implemented with PyTorch (v1.13.1) and MONAI (v1.2.0). Model training and testing were executed on an 8-core system (2.7 GHz) with 128 GB memory and an Nvidia V100 GPU cluster. To mitigate class imbalance, higher weight was assigned to the minority class (resolving nodules), while lower weight was assigned to the majority class (non-resolving nodules). These class weights were applied to the loss function during training. The model was trained using the Adam optimizer with a learning rate of 1e-4 for 100 epochs and a batch size of 4. Models were mainly selected based on the lowest validation loss. Heatmap was generated by GradCAM ++ (package grad-cam v1.4.8).

## 4 Participant-based calculation rule regarding DL outcome of nodule status

For participants with only one new intermediate nodule, the calculation aligns with the nodule-based approach. However, for participants with multiple nodules, the following rules applied:

1 True Negative: If a participant has at least one non-resolving nodule, and the model predicts a non-resolving outcome for at least one of these nodules, this case should be considered a true negative, regardless of other nodules in the participant.

2 True Positive: If a participant has only resolving nodules, and the model predicts resolving outcomes for all nodules, this case should be considered a true positive.

3 False Positive: If a participant has at least one non-resolving nodule, but the model predicts resolving outcomes for all nodules, this case should be considered a false positive.

4 False Negative: If a participant has only resolving nodules, but the model predicts at least one non-resolving nodule, this case should be considered a false negative.

## Appendix B: Results based on the dataset excluding ten nearly-resolved nodules

Table B1 Initial characteristics of new intermediate nodules by resolving the status

| | Non-resolving nodules (n=281) | Resolving nodules (n=53) | Nearly-resolved nodules (n=10) | P-value |
|---|---|---|---|---|
| **Volume** | | | | 0.65 |
| Median (IQR) | 127.6 (82.1, 236.9) | 135.6 (77.8,188.1) | 86.4 (77.2,179) | |
| **Maximum diameter** | | | | 0.78 |
| Median (IQR) | 8.4 (6.9,11) * | 8.4 (6.9,9.7) | 8.3 (7.3,9.7) † | |
| **Minimum diameter** | | | | 0.99 |
| Median (IQR) | 5.3 (4.4,6.9) * | 5.6 (4.5,6.7) | 5.2 (4.8,6.7) † | |

Note- Data are medians; data in parentheses are IQRs. P values are calculated using the Kruskal-Wallis tests for the three nodule groups.

* The medians and IQR calculations were based on 278 nodules because 3 nodules lacked maximum and minimum diameter.

† The medians and IQR calculations were based on 8 nodules because 2 nodules lacked maximum and minimum diameter.

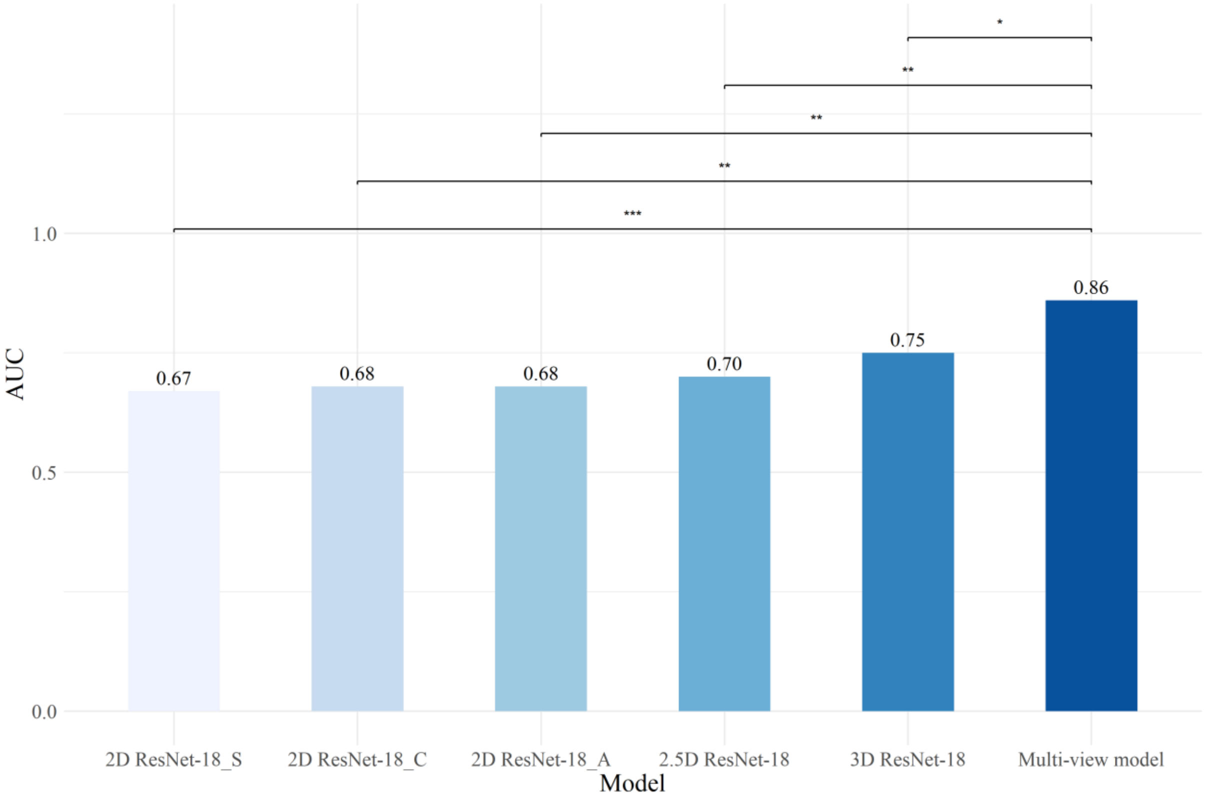


Figure B1 ROC comparison of models using the dataset excluding ten nearly-resolved nodules (our multi-view model is built on three 2D models and one 3D model; A: axial view; C: coronal view; S: sagittal view; *: significant at p < 0.05; **: significant at p < 0.01; ***: significant at p < 0.001)

Table B2 Sensitivity and specificity of 4-fold cross-validation

| | Sensitivity | Specificity |
|---|---|---|
| 2D ResNet-18_A | 0.45 ±0.11 | 0.83 ±0.06 |
| 2D ResNet-18_C | 0.47 ±0.07 | 0.84 ±0.07 |
| 2D ResNet-18_S | **0.55** ±0.11 | 0.79 ±0.11 |
| 2.5D ResNet-18 | 0.52 ±0.14 | 0.80 ±0.15 |
| 3D ResNet-18 | 0.53 ±0.13 | <u>0.87</u> ±0.03 |
| Multi-view model | <u>0.54</u> ±0.03 | **0.90** ±0.01 |

Note - our proposed multi-view model is built on three 2D models and one 3D model. A: axial view; C: coronal view; S: sagittal view; the best result is bolded, and the second-best result is underlined.

Table B3 Model prediction outcome based on participant level

| | True negative | False positive | True positive | False negative | Sensitivity | Specificity |
|---|---|---|---|---|---|---|
| Participant level | 201 | 13 | 22 | 14 | 0.61 | 0.94 |
